# A Novel Feature Selection Strategy Based on Salp Swarm Algorithm for Plant Disease Detection

**DOI:** 10.34133/plantphenomics.0039

**Published:** 2023-05-11

**Authors:** Xiaojun Xie, Fei Xia, Yufeng Wu, Shouyang Liu, Ke Yan, Huanliang Xu, Zhiwei Ji

**Affiliations:** ^1^ College of Artificial Intelligence, Nanjing Agricultural University, Nanjing, Jiangsu 210095, China.; ^2^ Center for Data Science and Intelligent Computing, Nanjing Agricultural University, Nanjing, Jiangsu 210095, China.; ^3^ State Key Laboratory for Crop Genetics and Germplasm Enhancement, Bioinformatics Center, Academy for Advanced Interdisciplinary Studies, Nanjing Agricultural University, Nanjing, Jiangsu 210095, China.; ^4^ Academy for Advanced Interdisciplinary Studies, Nanjing Agricultural University, Nanjing, Jiangsu 210095, China.; ^5^ Department of the Built Environment, College of Design and Engineering, National University of Singapore, 4 Architecture Drive, Singapore 117566, Singapore.

## Abstract

Deep learning has been widely used for plant disease recognition in smart agriculture and has proven to be a powerful tool for image classification and pattern recognition. However, it has limited interpretability for deep features. With the transfer of expert knowledge, handcrafted features provide a new way for personalized diagnosis of plant diseases. However, irrelevant and redundant features lead to high dimensionality. In this study, we proposed a swarm intelligence algorithm for feature selection [salp swarm algorithm for feature selection (SSAFS)] in image-based plant disease detection. SSAFS is employed to determine the ideal combination of handcrafted features to maximize classification success while minimizing the number of features. To verify the effectiveness of the developed SSAFS algorithm, we conducted experimental studies using SSAFS and 5 metaheuristic algorithms. Several evaluation metrics were used to evaluate and analyze the performance of these methods on 4 datasets from the UCI machine learning repository and 6 plant phenomics datasets from PlantVillage. Experimental results and statistical analyses validated the outstanding performance of SSAFS compared to existing state-of-the-art algorithms, confirming the superiority of SSAFS in exploring the feature space and identifying the most valuable features for diseased plant image classification. This computational tool will allow us to explore an optimal combination of handcrafted features to improve plant disease recognition accuracy and processing time.

## Introduction

The number of plant diseases caused by various bacterial, fungal, and viral infections has increased in recent years [1]. Plant diseases can cause substantial losses in agricultural production, resulting in economic losses [2]. Thus, timely and accurate detection of plant diseases is crucial for plant protection [3]. Although plant disease symptoms are evident in various organs of the plant [4], plant leaves are primarily used to detect infections [5,6]. The use of computer vision in plant disease detection has been gaining significant attention in the past few years due to its potential to accurately and efficiently detect plant diseases. Computer vision techniques involve using algorithms and machine learning (ML) models to analyze images of plant leaves and identify disease symptoms [7,8]. The use of computer vision in plant disease detection can enable early disease detection, which is critical for disease management and prevention.

High-throughput phenomics allows rapid, accurate, and high-throughput analysis of plant traits, including disease severity on a larger scale [9,10]. However, there is still a great demand for reliable and efficient computational methods/pipelines to address plant phenomics [11]. Developing novel phenotypic image-based algorithms will help to detect and classify plant disease quickly and accurately.

In the field of computer vision, models or algorithms for image classification rely on extracting local or global features of images [12–16]. There are 2 ways of describing image features, handcrafted features and non-handcrafted features [17–19]. Handcrafted features mainly depend on the prior knowledge of experts and are often used together with traditional ML methods for object detection and image classification [20–22]. Representative handcrafted descriptors include gradient statistics and local intensity comparison- and local binary pattern (LBP)-based methods [23–25]. However, handcrafted feature-based models require expert-designed feature detectors, descriptors, and vocabulary building methods for feature extraction and representation [26]. The whole process is labor-intensive and requires relevant expertise [26–28]. Non-handcrafted features can be extracted using the compact binary descriptor (CBD), convolutional neural networks (CNN), or principal component analysis network (PCAN) [29–31]. In particular, CNN-based deep features are robust and efficient compared to the handcrafted features [18,29,32], due to their independence of prior knowledge and image extraction and applicability to any images. However, non-handcrafted features have the following limitations: (a) The interpretability of non-handcrafted features is low, which means that it is difficult to describe the learned features [33,34]. (b) It is hard to obtain large datasets for model training [35,36]. Therefore, developing a novel feature selection strategy can potentially identify important features and increase the classification accuracy.

There is little literature on feature selection-based plant disease detection and identification. Some studies manually select top-ranked image features for leaf disease detection using feature ranking strategies [37,38]. Recently, metaheuristic algorithms developed in light of biology have been used for efficient feature selection, but rarely for improving image-based plant disease detection [39].

In this study, we proposed an enhanced salp swarm algorithm for feature selection (SSAFS) in image classification of diseased plants. Firstly, for leaf images of diseased plant, we defined 171 handcrafted features uniformly, including 45 color features and 126 texture features. Secondly, SSAFS was applied on each dataset to screen the optimal subsets of handcrafted features. Finally, all the SSAFS-derived potential feature subsets were evaluated with a neural network classifier. During the experiments, we applied SSAFS on 4 datasets from the UCI machine learning repository [40] and verified the performance of the developed method. SSAFS was further tested on 6 phenomics datasets of diseased plants obtained from PlantVillage [7]. Our results demonstrated that SSAFS not only significantly reduces the count of features, but also significantly improves the classification accuracy. Comparison analysis further shows that SSAFS outperforms multiple state-of-the-art (SOTA) methods.

The main contributions of the paper are as follows:

• This is the first study that develops a swarm intelligence algorithm (SIA) for image-based plant disease detection and severity grading estimation.
• An enhanced SSAFS was developed for identifying the valuable handcrafted features of plant images.
• Five kinds of well-known SIA models for feature selection were compared. The feature subset obtained from the SSAFS model achieved the highest classification accuracy.

The rest of this article is organized as follows: The Related Work gives a brief description of the related works. Details of the SSAFS algorithm, simulation experiment are described in Materials and Methods. Experimental results are presented and analyzed in Results. Discussion provides a discussion of the main findings. Finally, the Conclusion section provides the conclusion and future work.

## Related Work

Computer vision-based plant disease detection is an active area of research in the field of intelligent phytoprotection. In recent years, there has been growing interest in developing automated systems that can detect and diagnose plant diseases using computer vision techniques [6,41–43]. Building ML models for classification tasks is a traditional but effective approach. However, discovering, selecting, and understanding appropriate features are crucial for these classification models. This is related to whether the models perform well and whether the features are interpretable. Currently, there are 2 types of computational strategies available for the image-based classification of plant diseases: handcrafted feature-based methods and non-handcrafted feature-based methods.

### Handcrafted feature-based methods

Traditional ML models often use “handcrafted” features for object recognition and computer vision [35,44]. These features are manually designed by experts based on their observations to address specific issues, such as occlusions and changes in scale and illumination [17]. The most commonly used manual features include various morphological features, such as color, texture, and shape, to simulate the appearance of the object of interest [45,46]. So far, many algorithms based on handcrafted features have been applied for automatic plant disease recognition [47]. The most representative of these algorithms is the combination of morphological features extracted from leaf images and ML models (e.g., Support Vector Machine [SVM], artificial neural network ANN, K-Nearest Neighbors Algorithm [KNN], K-means, etc.) to achieve plant disease detection [48–54]. Although the handcrafted features are interpretable, it is unavoidable to mix in redundant and irrelevant features, which will affect the performance of ML models. Various variable selection methods are developed via filter-based strategies [55] to select the most robust features for plant image classification, including ReliefF [56], correlation-based methods [57,58], and minimal redundancy–maximal relevance [59]. The above filter strategies ignore feature dependencies, making removing redundant features difficult [60]. Unlike the filter models, wrapper methods use the prediction performance of the ML model as a metric to help search for the best subset of features [61]. It is worth mentioning that metaheuristic algorithms have been widely used for feature selection in computer vision in order to optimize feature dependencies and feature-classifier interactions [62–65]. The application fields of metaheuristic algorithms include Genetic Algorithm [54], Particle Swarm Optimization (PSO) [66–69], Squirrel search algorithm [70], and Artificial Bee Colony (ABC) [71,72]. However, the potential of these algorithms may be weakened when faced with high-dimensional problems [73]. Therefore, developing efficient and generalized feature selection algorithms for plant disease detection is still challenging. Overall, handcrafted feature-based methods have played an essential role in the field of plant phenomics processing. However, its obvious limitation is overdependence on expert knowledge.

### Non-handcrafted feature-based methods

Non-handcrafted features can be extracted in 3 ways: CNN [17], PCAN [74], and CBD [31]. Among them, CNN-based deep features are the most representative and have gradually superseded shallow classifiers trained using handcrafted features [17,19,20,47]. The advantages of CNN over traditional methods include hierarchical feature representation, exponentially improved expressive capability, and multi-task joint optimization [20]. Because of these advantages, CNN has been widely applied in image classification [19,75 ,76], face recognition [77–79], and video analysis [80–82] tasks. Similarly, CNN models are introduced to agricultural applications, e.g., disease recognition [7], weed detection [83], flower counting [84], and fruit grading [85]. One of the advantages of using CNNs for learning is that they are able to automatically learn and extract relevant features from raw input data, thereby eliminating the need for manual feature engineering [47]. In plant disease detection, acquiring large and diverse samples can be challenging due to many factors. Therefore, transfer learning provides an effective way in training CNN classifiers. Researchers have developed extensive CNN models and extracted deep features for image-based disease classification and severity grading of various plant diseases, including cucumber leaf disease [86,87], nutritional deficiencies and damage on apple leaves [88], banana and tomato leaf diseases [89,90], pest diseases on rice [91], and leaf spot disease in sugar beet [92]. However, the prediction accuracy provided by deep learning techniques depends on sufficient training samples. Moreover, when a training set does not have samples belonging to a certain class, the decision boundary may be overstrained [93]. In addition, the uninterpretable high dimensionality of deep features can lead to higher computational costs.

According to the above summary, we believe that there is still much space for improvement in both approaches. Therefore, in this study, we use a feature selection strategy to optimize the performance of handcrafted-based plant disease detection.

## SSAFS algorithm for Diseased Plant Classification

**Fig. 1.**
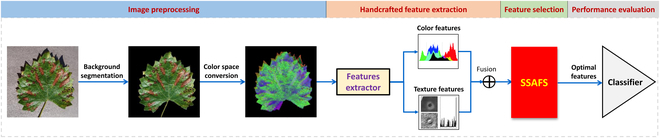
Image-based plant disease detection optimized by the SSAFS algorithm.

The SSAFS-derived feature selection for diseased plant classification includes 4 steps (Fig. 1) and will be explained in detail in the following subsections.

## Image preprocessing

Before feature extraction, image preprocessing is required. Firstly, we remove the background and edges from each raw image and keep only the effective area of leaf so that the extracted local features can characterize the lesions in the leaf image. In this study, we used GrabCut algorithm [94] to implement foreground segmentation (Fig. S1). Secondly, each image is further converted to 5 color spaces (RGB, HSV, Lab, YCrCb, and Luv) so that we can extract color features at image level from different color spaces.

## Handcrafted feature extraction of plant phenomics

In this study, we defined 2 types of handcrafted features for image classification of diseased plant, including color and texture features. Color features are mainly represented by color moments, including color first-order moment (average), color second-order moment (variance), and color third-order moment (skewness) [95,96]. Since each pixel has 3 color channels in one color space, the color moment of one image has 9 components to describe [97]. Therefore, 5 color spaces will provide 45 color features. Moreover, the texture descriptors include CLCM (color-level co-occurrence matrices) and LBP [98–100]. The texture descriptors in RGB, HSV, and Lab space are focused. In total, we have 36 GLCM (gray-level co-occurrence matrix)-based texture features (contrast, dissimilarity, correlation, and homogeneity) and 90 LBP-based features. There are 171 handcrafted features extracted from each leaf image with lesion.

## Feature selection with the SSAFS algorithm

Salp swarm algorithm (SSA), proposed by Mirjalili et al. [101], is a relative new metaheuristic algorithms [102,103]. The inspiration of SSA is the swarming behavior of the sea organism called salps. The salps are barrel-shaped, free-floating tunicates from the family of Salpidae. When navigating and foraging in the ocean, salps often float together in chains of salps [104]. The basic idea behind the SSA algorithm is to imitate the swarming behavior of salps in the deep oceans based on the salps chain. The salp at the head of the chain acts as the leader, and the following salps are followers [105]. Each individual represents a candidate solution for the targeted problem (food source).

**Fig. 2.**
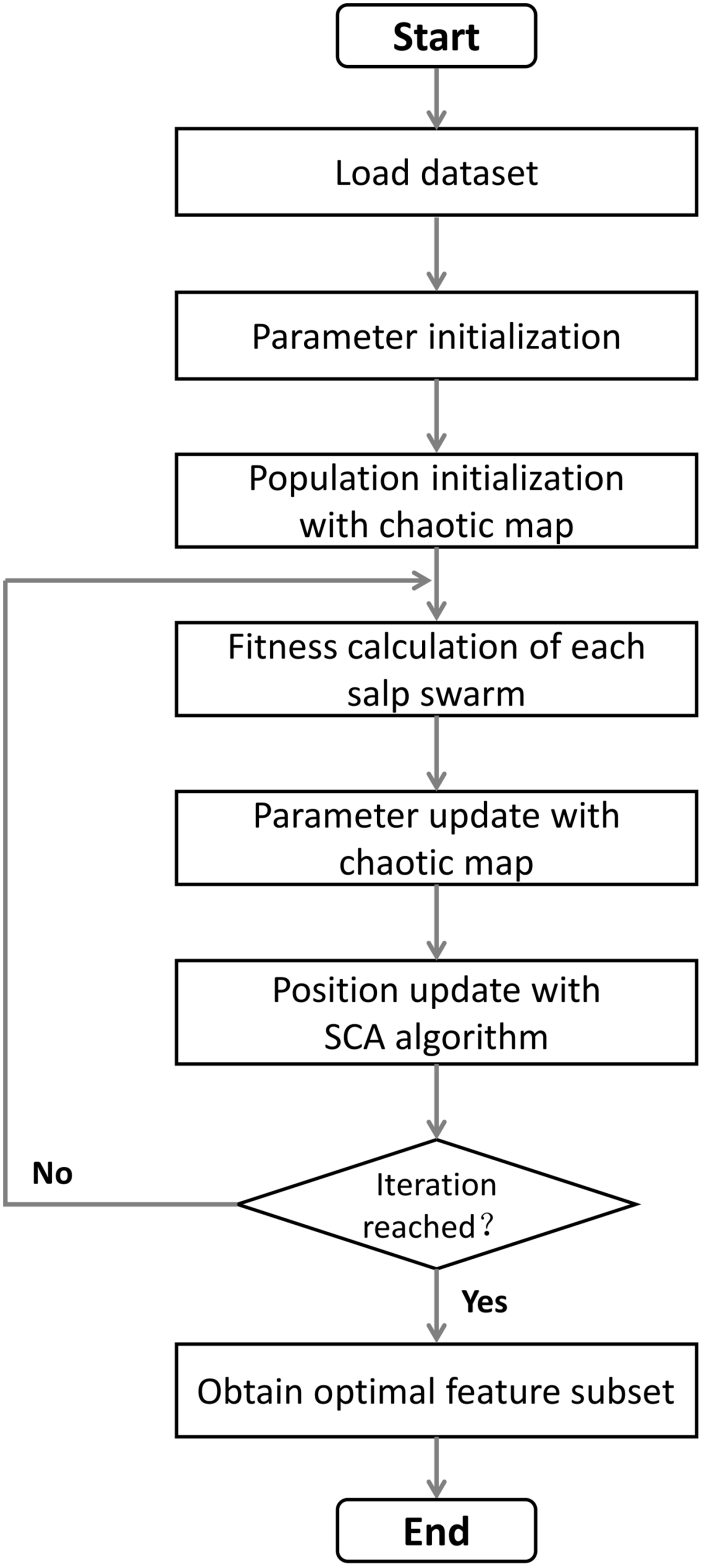
The flowchart of the proposed algorithm SSAFS.

A population of *N* salp individuals is defined as a 2-dimensional matrix $X=\left\{x_{1},\ldots ,x_{i},\ldots ,x_{N}\right\}=\left\{x_{j}^{i}|1\leq i\leq N,1\leq j\leq D\right\}$. $x_{j}^{1}$ denotes the position of the leader at the *j*th dimension. $x_{j}^{i}$ presents the position of the *i*th follower at the *j*th dimension (2 ≤ *i* ≤ *N*). When SSA algorithm is used for feature selection problems, all solutions are limited to the binary values, i.e., $x_{j}^{i}\in \left[0,1\right]$. $x_{j}^{i}$ represents the *j*th feature (color or texture) on the *i*th image sample. If $x_{j}^{i}=1$, the *j*th feature is selected. The feature selection framework based on the SSAFS algorithm is shown in Fig. 2.

**Fig. 3.**
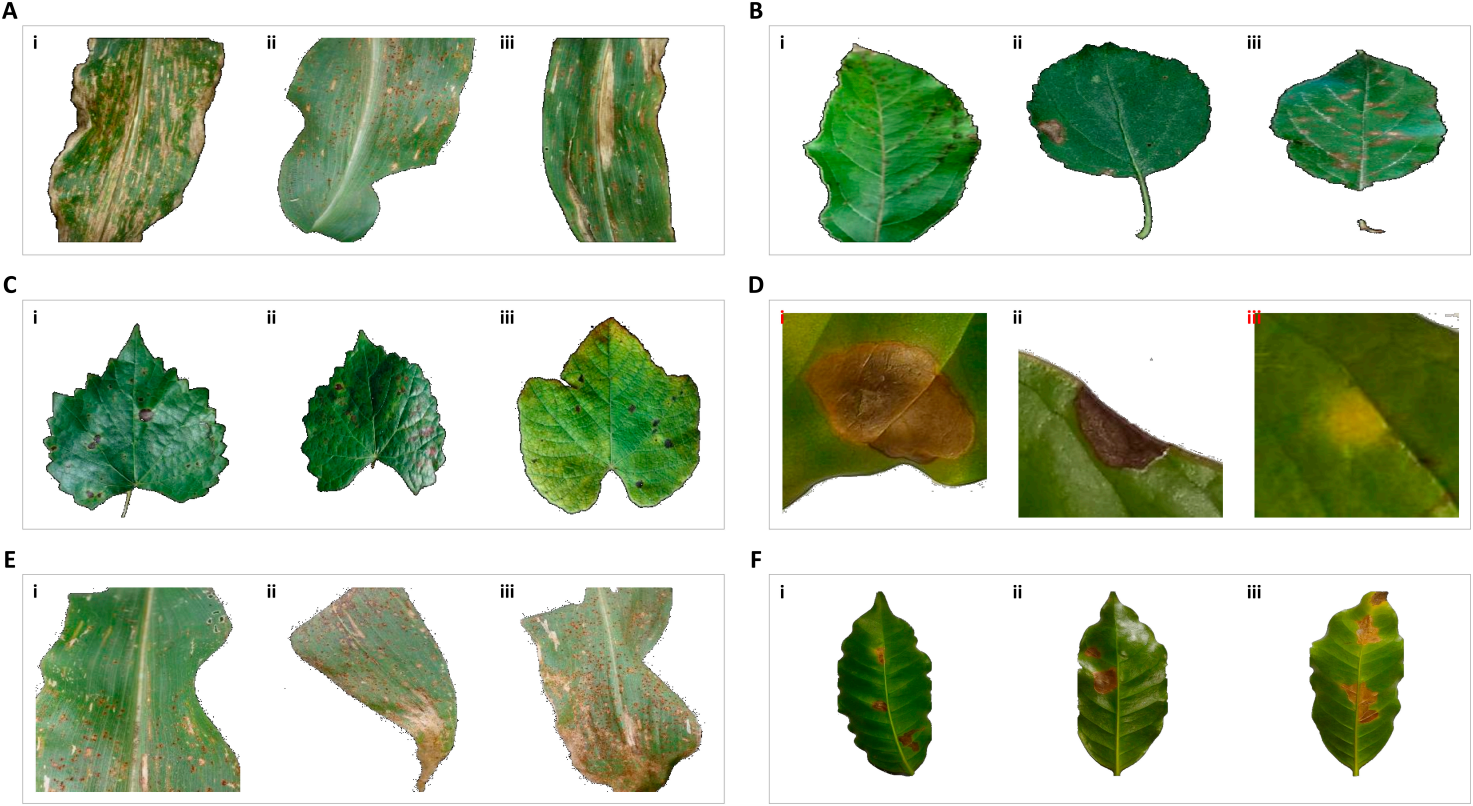
Phenotyping images of diseased plant are tested using the SSAFS approach. (A) Corn gray leaf spot, rust, and northern leaf blight. (B) Apple scab, black rot, and cedar apple rust. (C) Grape black rot, black measles, and leaf blight. (D) Coffee leaf miner, phoma, and rust. (E) Corn rust with early, mid, and late stages. (F) Coffee leaf miner with early, mid, and late stages.

### Population initialization

As shown in Fig. 2, the first step of SSAFS is population initialization. In this step, a swarm of *N* salp individuals is randomly generated. The quality of initial population is closely related to the convergence speed of the algorithm [106]. The chaotic map is a nonlinear dynamic system that could generate random numbers with special dynamics characteristics [107]. The generated random numbers exhibit non-repetitiveness, ergodicity, regularity, and unpredictability [108].

In our study, chaotic map with ergodic property [108] is employed to initialize uniform distributed salp to improve solutions diversity. In the original SSA, the initial state of the *i*th salp is defined as Eq. 1.

$$x_{j}^{i}\left(k\right)=\left({\mathrm{ub}}_{j}\left(k\right)-{\mathrm{lb}}_{j}\left(k\right)\right)*o^{k}+{\mathrm{lb}}_{j}\left(k\right)$$ (1)

where $x_{j}^{i}\left(k\right)$ represents the position of the *i*th follower in the *j*th dimension space at the iteration *k*; *ub_j_*(*k*) and *lb_j_*(*k*) denote the upper and lower bounds of the *j*th dimension, respectively. Equation 2 describes the expression of the logistic mapping for *o^k^*:

$$o^{k+1}=\mu o^{k}\left(1-o^{k}\right)$$ (2)

where *μ* is the bifurcation parameter of the logical mapping. Considering the fact that feature selection is a binary question, we need to define a transfer function to make the binary version of SSA from the continuous version [109,110]. Therefore, the variable $x_{j}^{i}\left(k\right)$ shown in Eq. 1 will be further transferred as Boolean state through the following Equations 3 and 4:

$$x_{j}^{i}\left(k\right)=\left\{\begin{array}{c} 1,T\left.\left(x_{j}^{i}\left(k\right)\right)\right)\geq C\\ 0,T\left(x_{j}^{i}\left(k\right)\right)<C \end{array}\right.$$ (3)

$$T\left(x_{j}^{i}\left(k\right)\right)=\frac{1}{1+\exp^{-x_{j}^{i}\left(k\right)}}$$ (4)

In Equation 3, $x_{j}^{i}\left(k\right)$ is equal to 1 if the *j*th feature in the *i*th individual is selected; otherwise, $x_{j}^{i}\left(k\right)=0$.

### Fitness calculation

In SIAs, the fitness function is an important metric to evaluate the strengths of individuals within a population [111]. The fitness value reflects the goodness of fit of each candidate's solution to the targeted question [112,113]. Therefore, the selection of the fitness function determines the balance of the multi-objective algorithm in the optimization process.

As a multi-objective problem, features selection try to minimize the subset of selected features and maximize the accuracy of the output for a given classifier, simultaneously [114]. According to the above basis, the fitness function for determining solutions in this situation built to achieve a balance between 2 objectives is defined in Eq. 5.

$$\text{Fitness}=\text{ρErr}\left(\mathrm{FS}\right)+\phi \frac{|\mathrm{FS}|}{N}$$ (5)

where the function *Err*(*) denotes the classification error of the potential feature subset ***FS***, and ∣***FS***∣ and *N* denote the number of selected features and the total number of features. The coefficients *ρ* and *ϕ* are the balance parameters that control the classification accuracy and the rate of features being selected. In addition, *ρ* and *ϕ* satisfy that *ρ* ∈ [0, 1] and *ρ* + *ϕ* = 1. A smaller fitness value is better.

*k*-NN algorithm is a non-parametric supervised learning algorithm. It relies on the closest *k* labeled instances (neighbors) to learn a function that produces an appropriate prediction for a given unlabeled example [115]. Here, the *k*-NN model was employed as the classification method to evaluate the feature subsets generated by SSAFS, where *k* = 3. Specifically, we use 5-fold cross-validation of *k*-NN to calculate the value *Err*(∗) of a feature subset.

### Population Evaluation

The role of the leader of the salp chain is to search for the food source. Hence, the position of the leader is dynamically updated based on the location of the food source. In the original SSA, the leader is updated by using the following Eq. 6:

$$x_{j}^{1}\left(k\right)=\left\{\begin{array}{c} F_{j}\left(k\right)+c_{1}*\left[\left({\mathrm{ub}}_{j}\left(k\right)-{\mathrm{lb}}_{j}\left(k\right)\right)*c_{2}+{\mathrm{lb}}_{j}\left(k\right)\right],c_{3}\geq 0.5\\ F_{j}\left(k\right)-c_{1}*\left[\left({\mathrm{ub}}_{j}\left(k\right)-{\mathrm{lb}}_{j}\left(k\right)\right)*c_{2}+{\mathrm{lb}}_{j}\left(k\right)\right],c_{3}<0.5 \end{array}\right.$$ (6)

where $x_{j}^{1}\left(k\right)$ denotes the position of the leader in the *j*th dimensional space in the *k*th iteration, and $F_{j}^{\left(k\right)}$ represents the position of the food source. $ub_{j}^{\left(k\right)}$ and $lb_{j}^{\left(k\right)}$ are the upper and lower boundaries, respectively. Parameters *c*_2_ and *c*_3_ control the step size and the directions of the next move, respectively (*c*_2_, *c*_3_ ∈ [0, 1]). Specifically, *c*_1_ is defined for balancing global search and local search capabilities:

$$c_{1}=2e^{-{\left(\frac{4k}{K}\right)}^{2}}$$ (7)

where *k* is the index of the current iteration, and *K* is the maximum of iterations. From Eq. 6, we found that the position of leader is mainly determined by the position of the current optimal food source. Once the leader falls into a local optimum, the whole population will fall into local stagnation.

To avoid the above issue, we introduce the Sine Cosine Algorithm (SCA) [116] here to improve the strategy of population evolution. The leader in our algorithm is updated through Eqs. 8 and 9:

$$x_{j}^{1}\left(k+1\right)=\left\{\begin{array}{c} F_{j}\left(k\right)+r_{1}*\sin\left(r_{2}\right)*\left|r_{3}*F_{j}\left(k\right)-x_{j}^{1}\left(k\right)\right|,r_{4}<0.5\\ F_{j}\left(k\right)-r_{1}*\cos\left(r_{2}\right)*\left|r_{3}*F_{j}\left(k\right)-x_{j}^{1}\left(k\right)\right|,r_{4}\geq 0.5 \end{array}\right.$$ (8)

$$r_{1}=a-k\frac{a}{K}$$ (9)

In Eq. (8), *r*_2_ is a parameter located in the interval [0,2π], which determines the neighborhood around the current solution in the search space. The coefficient *r*_3_ regulates the speed of the search process. *r*_4_ is used to switch the updating strategy of the leader between the sine and cosine components. In particular, *r*_1_ is a random number uniformly distributed in the range [0,1] that determines the direction of the current solution. In the early stage of the iterations, *r*_1_ helps to explore the search space, while contributing to exploiting the available search space.

Furthermore, the state of the follower was defined as follows:

$$x_{j}^{i}\left(k\right)=\frac{1}{2}ak^{2}+v_{0}k$$ (10)

where *v*_0_ is the initial velocity. Coefficient *a* is the acceleration and *a* = *v_final_*/*v*_0_. Overall, the location of the *i*th follower in the next iteration is jointly determined by its current and previous position:

$$x_{j}^{i}\left(k+1\right)=\frac{1}{2}\left(x_{j}^{i}\left(k\right)+x_{j}^{i-1}\left(k\right)\right)$$ (11)

## Classification performance evaluation based on an ANN

After obtaining the optimal feature subset through the SSAFS algorithm, we constructed an ANN model to investigate the performance for image classification. As shown in Fig. S2, there are 4 layers involved in this network, including an input layer, an output layer, and 2 *hidden* layers. The number of neurons in the input layer equals the dimensionality of an optimal feature subset obtained from SSAFS. The output layer consists of 3 neurons with a Boolean state, which indicates the discriminative results of image classification.

## Materials and Methods

### Data collection

In the experiments, we first applied the SSAFS approach to 4 UCI datasets, including Heart, Urban Land Cover, Arrhythmia, and CNAE-9 (Table 1). Secondly, we collected a set of diseased leaf images of apples, corn, grapes, and coffee from PlantVillage [7]. Based on the annotation information, we categorized these phenomics data into 2 groups. The first group contains 4 datasets for testing the classification performance of a classifier for different diseases on the same crop (Table 2). The second group includes 2 datasets for testing the classification (severity grading) performance of a classifier for different severity levels of the same disease (Table 3).

**Table 1.** Four UCI datasets used in this study.

**Dataset**	**No. of samples**	**No. of features**	**Class**
Heart	303	13	2
Urban land cover	168	148	9
Arrhythmia	452	279	16
CNAE-9	1,080	857	9

**Table 2.** Detailed information of the phenotyping datasets of plant with 3 types of diseases.

**Dataset**	**Organism**	**Disease 1**	**No. of samples**	**Disease 2**	**No. of samples**	**Disease 3**	**No. of samples**
DS_corn	Corn	Gray leaf spot	400	Rust disease	400	Northern corn leaf blight	400
DS_apple	Apple	Scab	250	Black rot	250	Cedar apple rust	250
DS_grape	Grape	Black rot	800	Black measles	800	Leaf blight	800
DS_coffee	Coffee	Leaf miner	413	Phoma leaf spot	351	Rust disease	689

**Table 3.** Detailed information of the phenotyping datasets of plant with different severities.

**Dataset**	**No. of sample (early stage)**	**No. of sample (mid stage)**	**No. of sample (late stage)**	**Disease**
DS_cn_rust	400	400	400	Rust disease
DS_cf_miner	237	65	19	Leaf miner

### Experiment design

We applied SSAFS to 4 UCI datasets with different scales to verify its effectiveness. We then further tested SSAFS on 6 plant phenomics datasets. After obtaining the optimal feature subset of each dataset, a neural network classifier was constructed to test its prediction power. The training set and testing set were assigned as 4:1. Moreover, we further evaluate if SSAFS provides faster convergence and stronger robustness. We run our method with 30 replicates to avoid the impact of population initialization on the model output. Finally, we selected PSO [117], ABC [118], Improved Binary Grey Wolf Optimization (IBGWO) [119], Squirrel search algorithm (abbreviated as “Squirrel”) [120], and standard SSA [101] as baseline algorithms to implement comparison analysis, which potentially reveals the advantages of our method.

### Parameter optimization

All the simulations were performed by using Python 3.6 and IDE Jupyter notebook under the environment with Ubuntu 18.04 on 6×Xeon E5-2678 v3 and 128 GB RAM. In the SSAFS framework, we set the iteration *K* as 50. Other parameters were set as follows: *μ* = 4, *c*_1_ = 6, *ρ* = 0.9, and *a* = 2. For the ANN classifier, the number of neurons in the latent layers is 128 and 32, respectively. Sigmod was used as the activation function in the output layer. The number of epochs in the ANN model is 200. In the comparison analysis, the default parameters are presented in Table 4.

**Table 4.** Parameter setting.

**Algorithms**	**Parameter values**
*SSAFS*	*c*_2_ and *c*_3_ random numbers over [0,1] are set as the literature [7]
*SSA*	*c*_2_ and *c*_3_ random numbers over [0,1] are set as the literature [7]
*ABC*	Limit = 100, Lower bound = 1; Upper bound = *N*
*PSO*	*c*_1_ = *c*_2_ = 1.49618*, w* = 0.7298
*IBGWO*	*m* = 0.99*, n* = 0.01
*Squirrel*	*N_fs_* = 4*, G_c_* = 1.9*, P_dp_* = 0.1

### Performance evaluation

In this study, the fitness score shown in Eq. 5 is defined to evaluate the goodness of any potential optimal feature subset. Accuracy represents the classification performance of a feature subset on a classifier. The dimensionality for a feature subset reflects how many important features potentially contribute to classification.

## Results

### SSAFS works well on UCI datasets

Table 5 shows the average performance of SSAFS and the other 5 SOTA algorithms on 4 UCI datasets. Due to the low dimensionality of the Heart dataset, the classification performance and the number of relevant features obtained by the 6 algorithms are very close. On the dataset Urban Land Cover, SSAFS significantly outperformed other algorithms. For CNAE-9 and Arrhythmia, SSAFS is close to IBGWO and SSA, but is superior to PSO, ABC, and Squirrel. However, the processed dataset is still high-dimensional after feature selection. We found that SSAFS can achieve higher classification accuracy with fewer features. Moreover, we examined the quality of the best solution provided by each algorithm to see the difference in global search. Table 6 indicates that the optimal solution found by SSAFS is better than other comparison methods.

**Table 5.** Performance comparisons between SSAFS and 5 baseline algorithms on UCI datasets.

**Metric**	**Method**	**Heart**	**Urban land cover**	**Arrhythmia**	**CNAE-9**
Accuracy	PSO	0.8406±0.02	0.5576±0.05	0.6027±0.03	0.8303±0.03
	ABC	0.8404±0.03	0.5786±0.04	0.6302±0.02	0.8356±0.02
	IBGWO	0.8382±0.02	0.6131±0.05	0.6372±0.03	0.8343±0.02
	Squirrel	0.8251±0.03	0.5774±0.06	0.6106±0.03	0.8287±0.03
	SSA	0.8415±0.01	0.5922±0.03	0.6514±0.01	0.8520±0.01
	SSAFS	**0.8423±0.01**	**0.7118±0.06**	**0.6567±0.01**	**0.8713±0.01**
Fit. value	PSO	0.1821±0.01	0.4326±0.04	0.4591±0.03	0.2108±0.05
	ABC	0.1823±0.01	0.4201±0.03	0.4085±0.04	0.2019±0.03
	IBGWO	0.1819±0.01	0.3875±0.03	0.3588±0.03	0.1935±0.02
	Squirrel	0.1820±0.01	0.4201±0.04	0.3917±0.03	0.2033±0.03
	SSA	0.1817±0.01	0.4115±0.03	0.3602±0.01	0.1845±0.01
	SSAFS	**0.1807±0.01**	**0.2785±0.05**	**0.3396±0.01**	**0.1763±0.01**
No. of feature	PSO	5.11±0.45	72.90±2.22	139.60±23.8	451.31±29.7
	ABC	5.11±0.58	68.78±6.17	135.07±18.2	446.74±11.8
	IBGWO	5.12±0.55	64.13±7.87	95.61±12.7	409.25±15.1
	Squirrel	5.13±0.69	69.22±6.91	115.44±21.3	421.78±17.6
	SSA	5.09±0.61	67.25±6.05	129.60±11.2	439.83±9.72
	SSAFS	5.13±0.63	**28.21±14.02**	**60.65±27.64**	**418.83±7.96**

**Table 6.** Performance of optimal solutions obtained from SSAFS for 4 UCI datasets.

**Metric**	**Method**	**Heart**	**Urban land cover**	**Arrhythmia**	**CNAE-9**
Accuracy	PSO	0.8249	0.6012	0.6278	0.8539
	ABC	0.8446	0.6517	0.6412	0.8570
	IBGWO	0.8416	0.6607	0.6438	0.8565
	Squirrel	0.8383	0.6429	0.6350	0.8519
	SSA	0.8446	0.6699	0.6529	0.8656
	SSAFS	0.8446	**0.8241**	**0.6587**	**0.8805**
Fit. value	PSO	0.1961	0.4028	0.3776	0.1808
	ABC	0.1782	0.3601	0.3620	0.1785
	IBGWO	0.1810	0.3466	0.3501	0.1779
	Squirrel	0.1840	0.3506	0.3636	0.1836
	SSA	0.1782	0.3439	0.3572	0.1717
	SSAFS	0.1782	**0.1612**	**0.3154**	**0.1553**
No. of feature	PSO	5	65	119	423
	ABC	5	69	109	427
	IBGWO	5	61	85	418
	Squirrel	5	67	98	431
	SSA	5	69	125	435
	SSAFS	5	**5**	**23**	**409**

### SSAFS works well on the phenomics of diseased plant

SSAFS was first applied to 6 phenomics datasets of the diseased plant (Tables 2 and 3). As described in the Handcrafted feature extraction of plant phenomics section, all of the phenomics datasets share the common 171 handcrafted features. Table 7 indicates that the potentially important features (for each dataset) screened by SSAFS appear to provide the best classification performance in both sample stratification and severity estimation. Compared with other algorithms, the optimal solution output from SSAFS contains relatively fewer features. These differences are significant on the dataset DS_cn_rust. Secondly, we compared the best solution of each phenomics dataset obtained from each algorithm (Table 8). In datasets DS_grape and DS_coffee, SSAFS outputs a combination of ~30 features, which shows high classification accuracy. On average, the dimensionality of the optimal solutions for all the datasets is 31.8, which is obviously superior to PSO, ABC, and SSA.

**Table 7.** Performance comparisons between SSAFS and 5 baseline algorithms on 6 plant phenomics datasets.

**Metric**	**Method**	**DS_corn**	**DS_apple**	**DS_grape**	**DS_coffee**	**DS_cn_rust**	**DS_cf_miner**
Accuracy	PSO	0.7087±0.04	0.8021±0.02	0.8634±0.02	0.8015±0.03	0.7493±0.01	0.7250±0.06
	ABC	0.7029±0.02	0.8126±0.03	0.8729±0.02	0.7886±0.02	0.7505±0.01	0.7369±0.07
	IBGWO	0.7192±0.02	0.8160±0.03	0.8833±0.02	0.8004±0.03	0.7692±0.01	0.7601±0.03
	Squirrel	0.7108±0.02	0.8107±0.03	0.8821±0.02	0.7901±0.04	0.7517±0.01	0.7508±0.04
	SSA	0.7132±0.01	0.8075±0.02	0.8856±0.01	0.7961±0.01	0.7392±0.01	0.7464±0.01
	SSAFS	**0.7226±0.02**	**0.8133±0.02**	**0.8871±0.01**	**0.8046±0.01**	**0.7629±0.02**	**0.7691±0.01**
Fit. value	PSO	0.3097±0.03	0.2165±0.01	0.1533±0.01	0.2114±0.02	0.2677±0.01	0.2925±0.04
	ABC	0.2996±0.03	0.2020±0.02	0.1497±0.01	0.2353±0.01	0.2591±0.01	0.2747±0.05
	IBGWO	0.2878±0.01	0.1978±0.02	0.1389±0.01	0.2042±0.02	0.2481±0.01	0.2411±0.04
	Squirrel	0.3018±0.02	0.2055±0.02	0.1435±0.01	0.2223±0.02	0.2603±0.01	0.2553±0.04
	SSA	0.3025±0.01	0.2181±0.01	0.1446±0.01	0.2299±0.01	0.2793±0.01	0.2703±0.01
	SSAFS	**0.2786±0.02**	**0.1938±0.02**	**0.1219±0.01**	**0.2018±0.01**	**0.2232±0.01**	**0.2183±0.01**
No. of feature	PSO	74.91±7.53	66.45±5.90	52.93±7.81	56.72±8.56	72.29±3.52	78.10±9.91
	ABC	55.26±9.61	57.37±9.34	60.37±9.22	78.86±7.49	59.61±5.75	62.10±6.27
	IBGWO	60.25±6.79	51.10±6.27	58.41±8.26	41.65±5.94	65.89±7.40	42.95±4.35
	Squirrel	71.94±7.62	60.39±9.61	64.39±9.24	57.39±8.58	62.05±8.75	53.93±8.40
	SSA	75.80±5.25	77.83±5.05	71.43±6.68	80.33±6.52	76.76±8.02	73.10±5.92
	SSAFS	**49.96±14.48**	**44.40±10.73**	**35.10±14.38**	**45.26±15.52**	**17.16±10.9**	**19.26±12.75**

**Table 8.** Performance of optimal solutions obtained from SSAFS for 6 plant phenomics datasets.

**Metric**	**Method**	**DS_corn**	**DS_apple**	**DS_grape**	**DS_coffee**	**DS_cn_rust**	**DS_cf_miner**
Accuracy	PSO	0.7150	0.8175	0.8881	0.8209	0.7512	0.7706
	ABC	0.7025	0.8343	0.8950	0.8150	0.7622	0.8025
	IBGWO	0.7242	0.8360	0.8983	0.8204	0.7783	0.7975
	Squirrel	0.7158	0.8293	0.8963	0.8190	0.7717	0.7882
	SSA	0.7189	0.8211	**0.9092**	0.8175	0.7647	0.7718
	SSAFS	**0.7594**	**0.8491**	0.9087	**0.8285**	**0.7940**	**0.8031**
Fit. value	PSO	0.2968	0.2011	0.1352	0.1898	0.2672	0.2486
	ABC	0.2952	0.1778	0.1325	0.2080	0.2497	0.2117
	IBGWO	0.2810	0.1751	0.1214	0.1868	0.2334	0.2080
	Squirrel	0.2950	0.1846	0.1261	0.1916	0.2376	0.2175
	SSA	0.2910	0.2037	0.1273	0.2046	0.2515	0.2439
	SSAFS	**0.2370**	**0.1598**	**0.0956**	**0.1707**	**0.2041**	**0.1959**
No. of feature	PSO	69	63	59	49	74	72
	ABC	47	49	65	71	61	58
	IBGWO	56	47	51	43	58	44
	Squirrel	67	53	56	49	55	46
	SSA	65	73	78	69	68	66
	SSAFS	**35**	**41**	**23**	**28**	**32**	**32**

To further validate the performance of SSAFS, the ANN model is employed as the classifier to evaluate the optimal solutions searched by SSAFS. From Table 9, we find that the optimal feature subsets of the first 4 datasets output by SSAFS still have the highest classification accuracy on ANN models. Combining Figs. S3 and S4, we found that the improvement of SSAFS is relative to standard SSA. Finally, the computational cost of all the algorithms was evaluated on 6 plant datasets. From Table 10, we can easily find that SSAFS works efficiently on datasets with smaller samples. IBGWO also provides low computational costs on DS_coffee and DS_cn_rust. In summary, we suggest that the proposed SSAFS provides a new way to screen valuable handcrafted features for image classification of diseased plants.

**Table 9.** Performance of optimal solutions obtained from SSAFS on neural network for 6 plant phenomics datasets.

**Method**	**DS_corn**	**DS_apple**	**DS_grape**	**DS_coffee**	**DS_cn_rust**	**DS_cf_miner**
PSO	0.9275	0.9366	0.9328	0.9590	0.9210	0.8961
ABC	0.9167	0.9490	0.9517	0.9244	0.9359	0.9015
IBGWO	0.9208	0.9587	0.9558	0.9704	0.9258	0.9003
Squirrel	0.9133	0.9507	0.9454	0.9683	0.9308	0.9034
SSA	0.9424	0.9759	0.9606	0.9740	**0.9871**	**0.9209**
SSAFS	**0.9455**	**0.9795**	**0.9643**	**0.9747**	0.9465	0.9158

**Table 10.** Time complexity of 6 meta-heuristic methods on plant phenomics datasets (unit: S).

**Method**	**DS_corn**	**DS_apple**	**DS_grape**	**DS_coffee**	**DS_cn_rust**	**DS_cf_miner**
PSO	289.12	177.21	675.24	358.95	279.68	145.56
ABC	271.39	179.38	604.33	343.98	260.85	138.74
IBGWO	283.46	163.58	598.20	**318.85**	**241.05**	126.42
Squirrel	259.89	169.10	**586.71**	349.53	256.87	147.18
SSA	261.06	165.89	607.26	332.66	252.18	127.09
SSAFS	**251.30**	**160.62**	589.18	325.31	254.76	**124.73**

### The robustness of the SSAFS algorithm

**Fig. 4.**
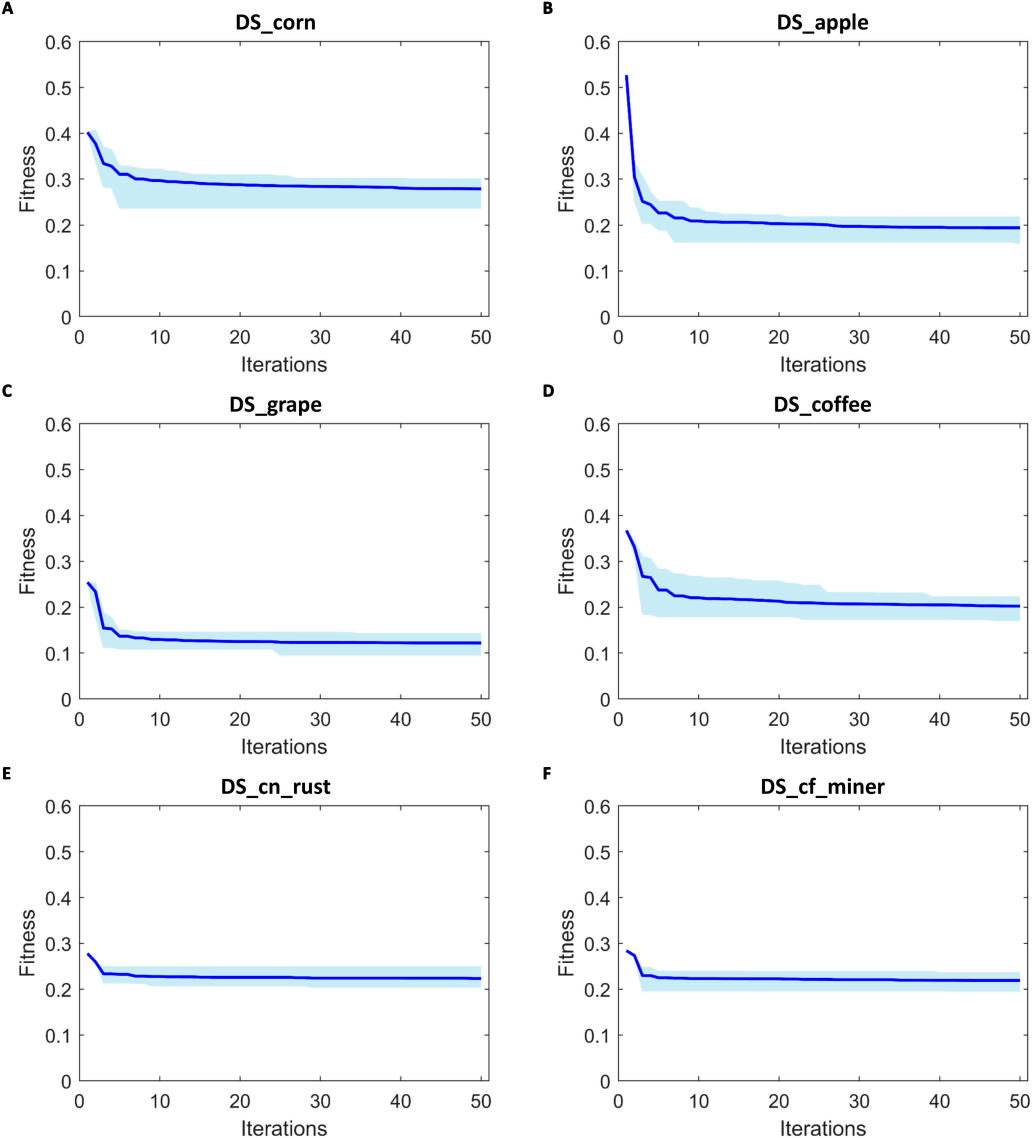
Comparison of convergence performance of SSAFS and SSA on 6 plant disease datasets. (A) Corn diseases. (B) Apple diseases. (C) Grape diseases. (D) Coffee diseases. (E) Three grades of corn rust. (F) Three grades of coffee leaf miner.

**Fig. 5.**
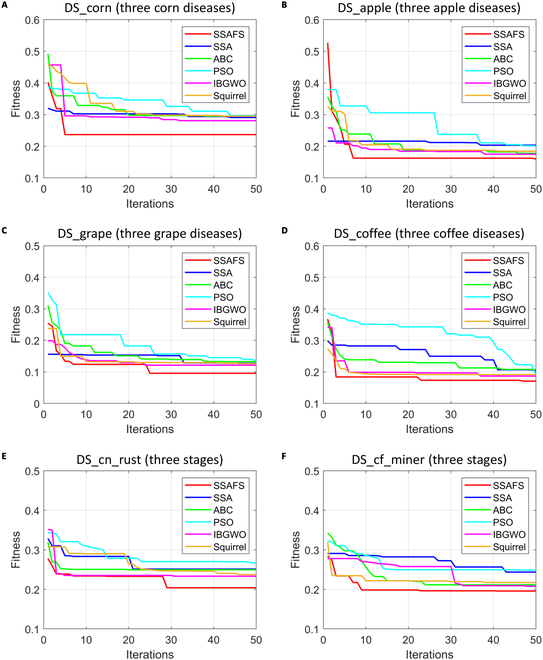
Stability analysis of SSAFS and 5 baseline methods on 6 plant disease datasets. (A) Corn diseases. (B) Apple diseases. (C) Grape diseases. (D) Coffee diseases. (E) Three grades of corn rust. (F) Three grades of coffee leaf miner.

To analyze the robustness, we further evaluated the proposed SSAFS method from 2 aspects. In one aspect, we checked if the converge curve of SSAFS was steady across multiple replicates. From Fig. 4, we find that SSAFS shows stable convergence on 5 datasets except for DS_coffee. It proves that population initialization has no significant impact on the output of SSAFS optimization. We also checked if the SSAFS algorithm quickly converges to the optimal solution in iterations. In Fig. 5, we can see that SSAFS not only provides a better solution than the other 3 algorithms but also converges significantly faster. In particular, SSAFS exhibited extremely fast convergence rates on dataset DS_corn (Fig. 5A) and DS_apple (Fig. 5B) and reached global solutions within 10 iterations. Figures S5 and S6 indicate that our method displays good convergence. Overall, we conclude that the proposed method is reliable for feature selection.

### Statistical analysis for the SSAFS-derived features of plant phenomics datasets

**Fig. 6.**
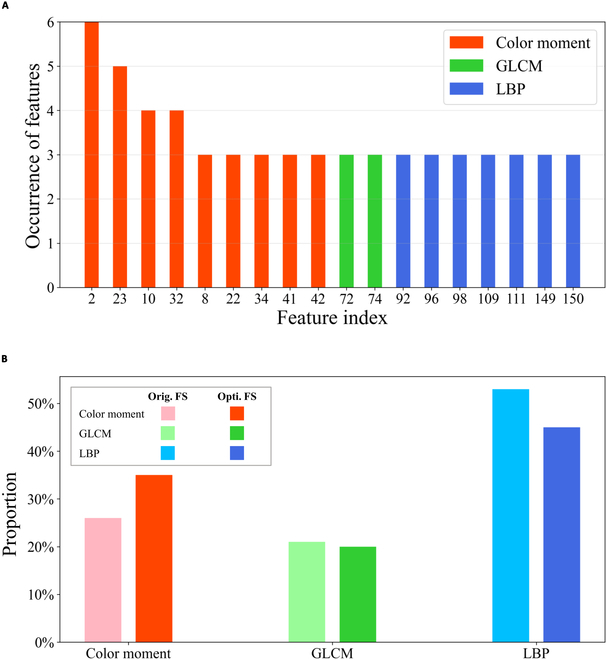
The analysis of the optimal features of 6 datasets obtained from the SSAFS algorithm. (A) Eighteen features occur in more than half of the optimal feature subsets. (B) The proportion of Color moment, CLCM, and LBP before and after feature selection.

In this section, we discuss the biological meaning of the potential features calculated by SSAFS. Of the 171 handcrafted features we defined above, 18 important features were present in the optimal feature subsets of at least 3 phenomics datasets (Fig. 6A). Among these 18 features, the proportion of color features is higher than that of texture features, indicating that color features play a more important role in plant image classification. In particular, we noticed that the top 4 color features come from the spaces RGB, LAB, HSV, and YCrCb (Table 11), including 3 variables for “variance” and 1 variable for “average” of color moment. It seems that variance has stronger stratification power rather than average and skewness.

**Table 11.** Frequency of several key features across 6 plant phenomics datasets.

**Index**	**Feature name**	**Category**	**Frequency on 6 datasets**	**Frequency on replicates**
2	Std_R	RGB	6/6	22.5/30
23	Std_A	LAB	5/6	11.4/30
10	Mean_H	HSV	4/6	11.75/30
32	Std_Cr	YCrCb	4/6	13.5/30

Moreover, we examined the proportions of color and texture features before and after feature selection. Combining 6 optimal feature subsets (derived by SSAFS) for all the phenomics datasets, we obtained 73 color features and 137 texture features (42 for GLCM and 95 for BLP). From Fig. 6B, it is evident that only the proportions of color features increase significantly after feature selection. Compared with CLCM, LBP-based features may include some irrelevant variables, which can be removed by SSAFS.

## Discussion

In this study, an enhanced SSAFS was developed to image classification of diseased plants. In the proposed method, using chaotic maps for population initialization can effectively improve the diversity of solutions. Specifically, SCA algorithm not only prevents the heuristic search from falling into local optimums, but also speeds up the convergence rate.

The proposed SSAFS algorithm was validated using 4 UCI public datasets and 6 phenomics datasets of diseased plants. The results were compared with 5 other heuristic feature selection techniques, namely, PSO, ABC, IBGWO, Squirrel, and SSA. The simulation results indicate that the performance of the SSAFS-based method is better than traditional wrapper feature-selecting techniques. The optimal solutions searched by SSAFS are less dependent on the classifier. More importantly, one of the crucial contributions of this work to plant phenomics is the definition of handcrafted features and the precision screen of relevant features through a novel computational approach. It provides new insight into computer vision-based plant image classification.

Limitations exist in the current work. Although our method shows great convergence and robustness, there is no guarantee that 30 replicates on new datasets are still reasonable. Therefore, we suggest that parallel computing should be assigned at least 100 replicates. Moreover, morphology and shape are also important features that were not considered in the current study. Since the classification accuracy mostly depends on the noise level within the images, verifying the proposed method in outdoor environments is also necessary. Furthermore, more extensive testing on various phenomics datasets will be necessary in the future.

## Conclusion

In this study, we proposed an SIA for feature selection (SSAFS). The SSAFS method was used to identify handcrafted features of diseased plant images to obtain classifiers with higher accuracy. Our approach outperforms other swarm intelligence methods in screening the most valuable features. Furthermore, our findings highlight the importance of local features that are critical for disease detection.

Future work will adopt other population-based multi-objective methods to deal with similar problems. We propose to combine comprehensive handcrafted and non-handcrafted features of plant images for accurate and efficient detection in the field of phenomics. In addition to leaf images, phenomics data collected from other plant organs are also valuable for plant disease detection.

## Data Availability

All the processed data and source code can be freely accessed at GitHub: https://github.com/JakeJiUThealth/SSAFS_V1.0.

## Supplementary Materials

Supplementary Materials Fig. S1. Grabcut algorithm for background segmentation of leaf images. Fig. S2. Neural network classifier for evaluating the optimal feature subsets. Fig. S3. Performance test of optimal solutions obtained from SSAFS on neural network for 6 plant phenomics datasets. Fig. S4. Performance test of optimal solutions obtained from SSA on neural network for 6 plant phenomics datasets. Fig. S5. The distribution of the optimal solutions in parallel computing on the UCI dataset. Fig. S6. The distribution of the optimal solutions in parallel computing on the plant phenomics dataset.
