# Supplementary material for: A Novel Feature Selection Strategy Based on Salp Swarm Algorithm for Plant Disease Detection
Source: Plant Phenomics. 2023 May 11;5:0039. doi: 10.34133/plantphenomics.0039 (PMC10204742; doi:10.34133/plantphenomics.0039)
Supplement: Supplementary Materials — Fig. S1. Grabcut algorithm for background segmentation of leaf images. Fig. S2. Neural network classifier for evaluating the optimal feature subsets. Fig. S3. Performance test of optimal solutions obtained from SSAFS on neural network for 6 plant phenomics datasets. Fig. S4. Performance test of optimal solutions obtained from SSA on neural network for 6 plant phenomics datasets. Fig. S5. The distribution of the optimal solutions in parallel computing on the UCI dataset. Fig. S6. The distribution of the optimal solutions in parallel computing on the plant phenomics dataset. [file plantphenomics.0039.f1.docx]

**Supplementary Figures**


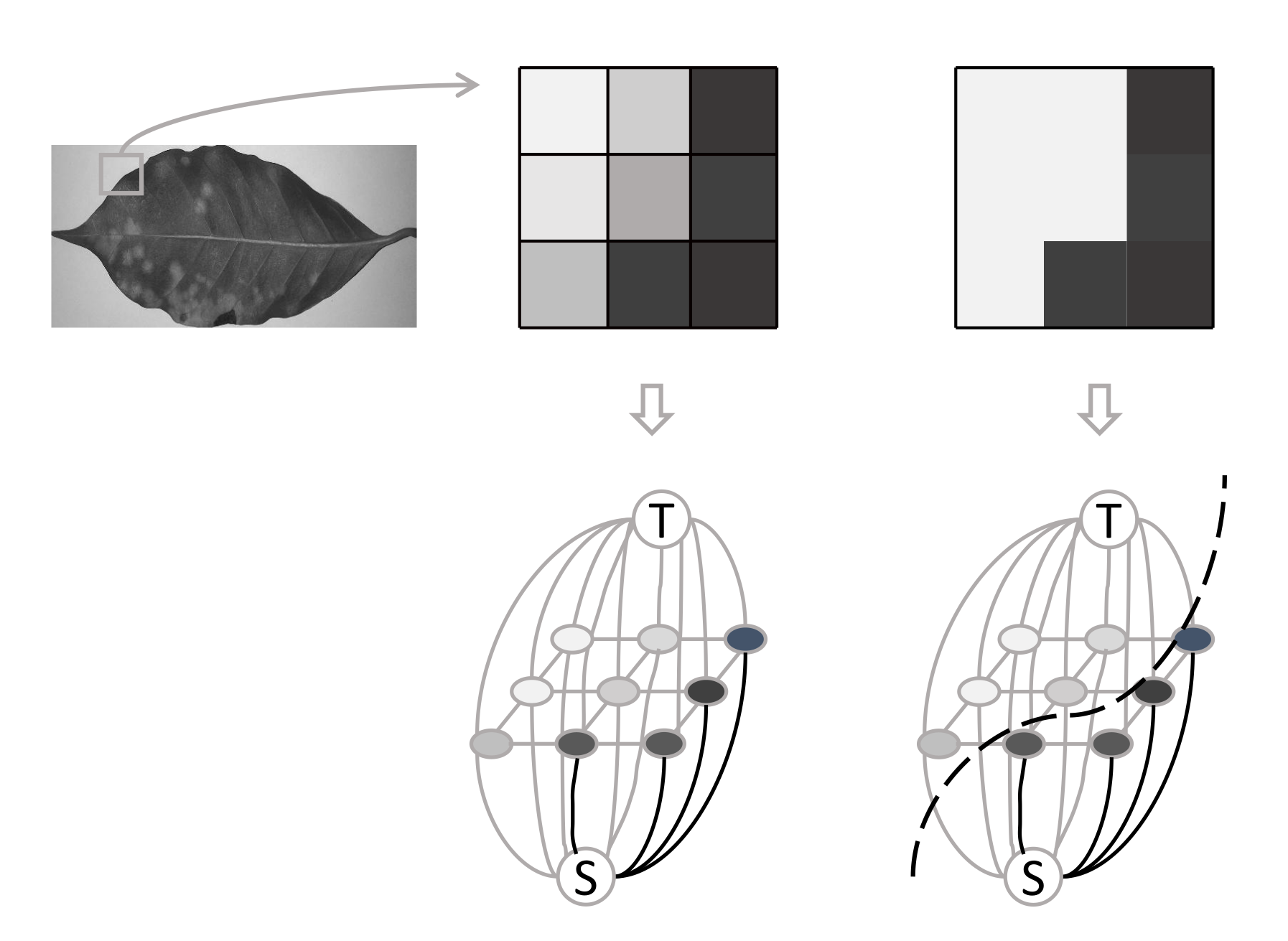


**Fig S1**. Grabcut algorithm for background segmentation of leaf images.


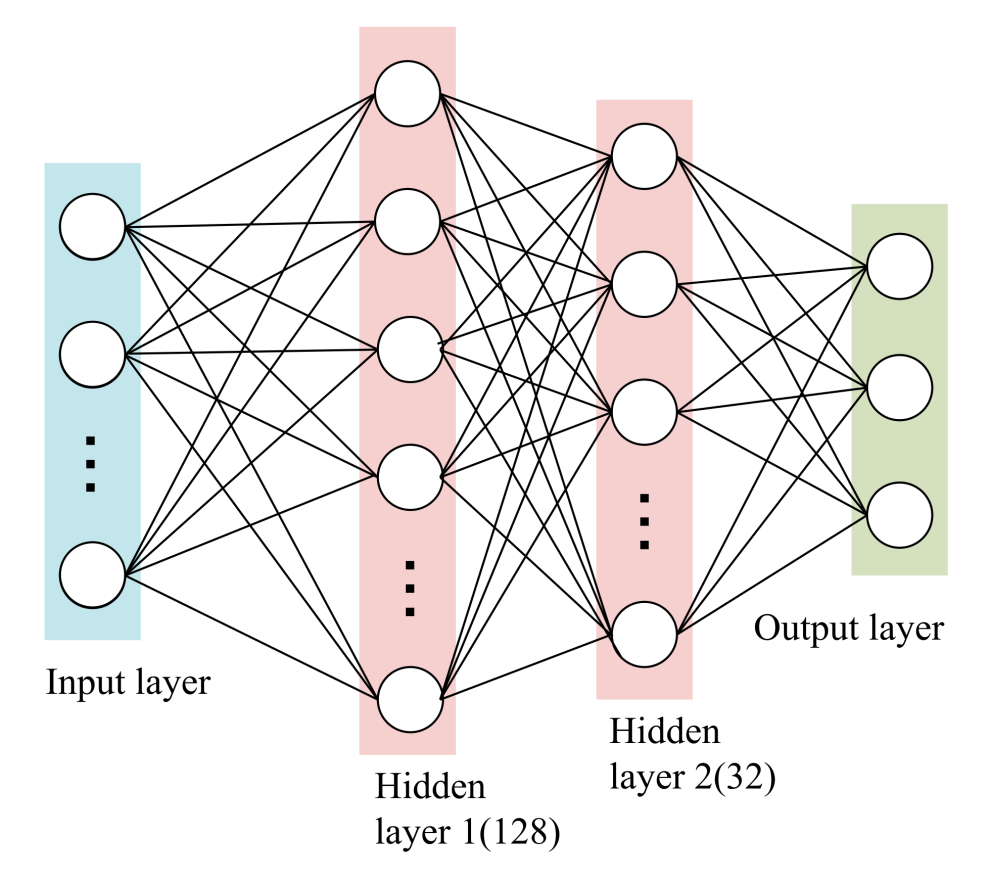


**Fig S2**. Neural network classifier for evaluating the optimal feature subsets.


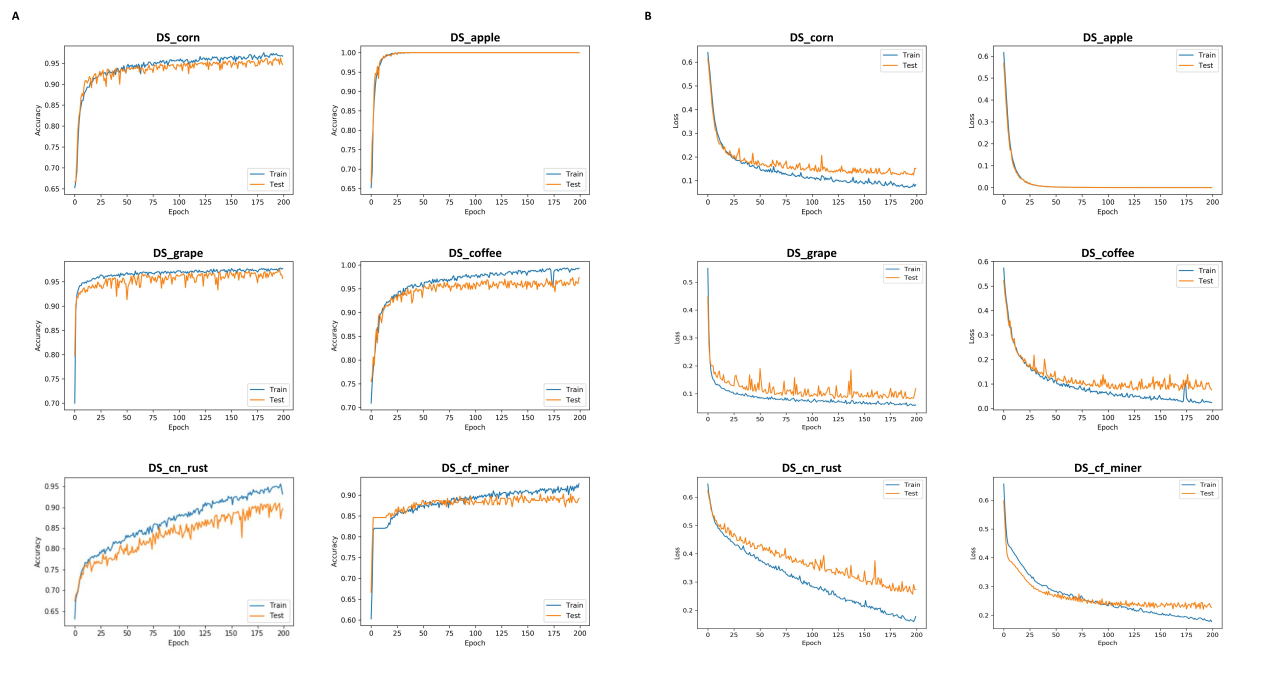


**Fig S3**. Performance test of optimal solutions obtained from SSAFS on neural network for six plant phenomics datasets. (A) Accuracy; (B) Loss


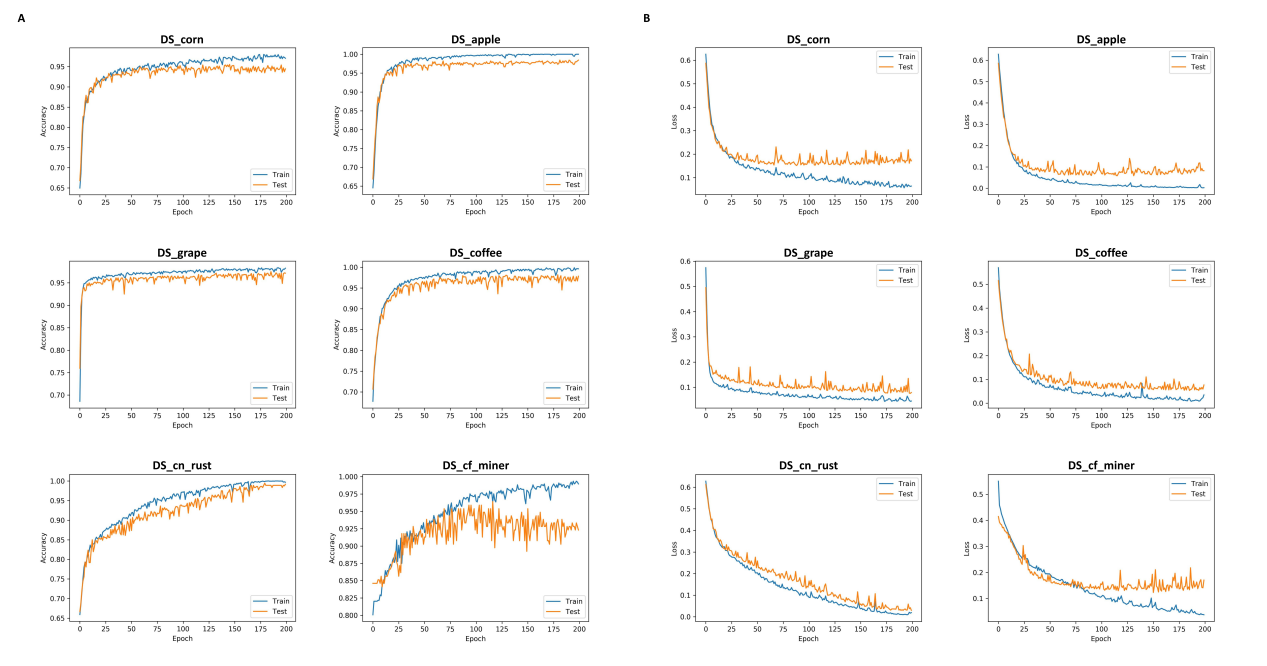


**Fig S4**. Performance test of optimal solutions obtained from SSA on neural network for six plant phenomics datasets. (A) Accuracy; (B) Loss


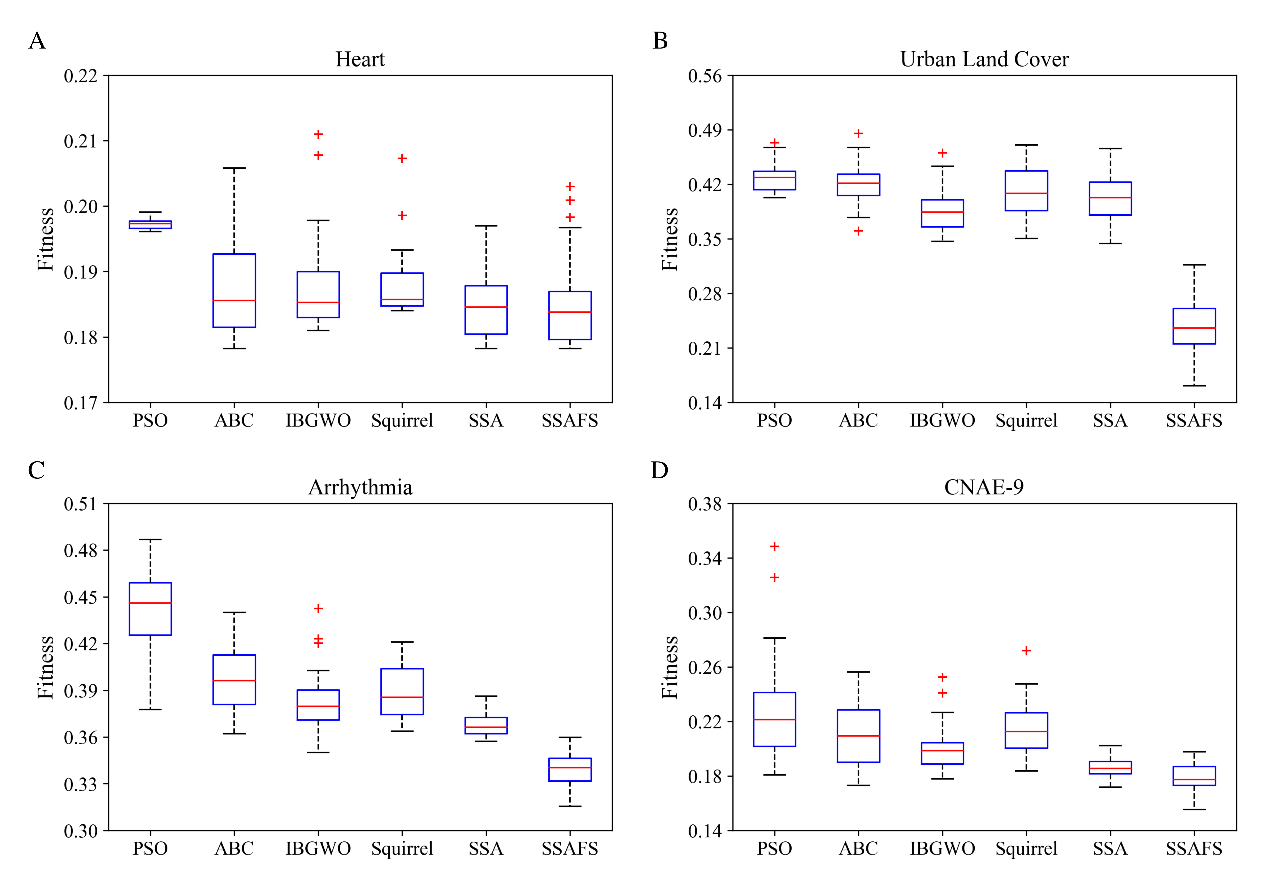


**Fig S5**. The distribution of the optimal solutions in the parallel computing on UCI dataset (A) Heart; (B) Urban Land Cover; (C) Arrhythmia; and (D) CNAE-9.


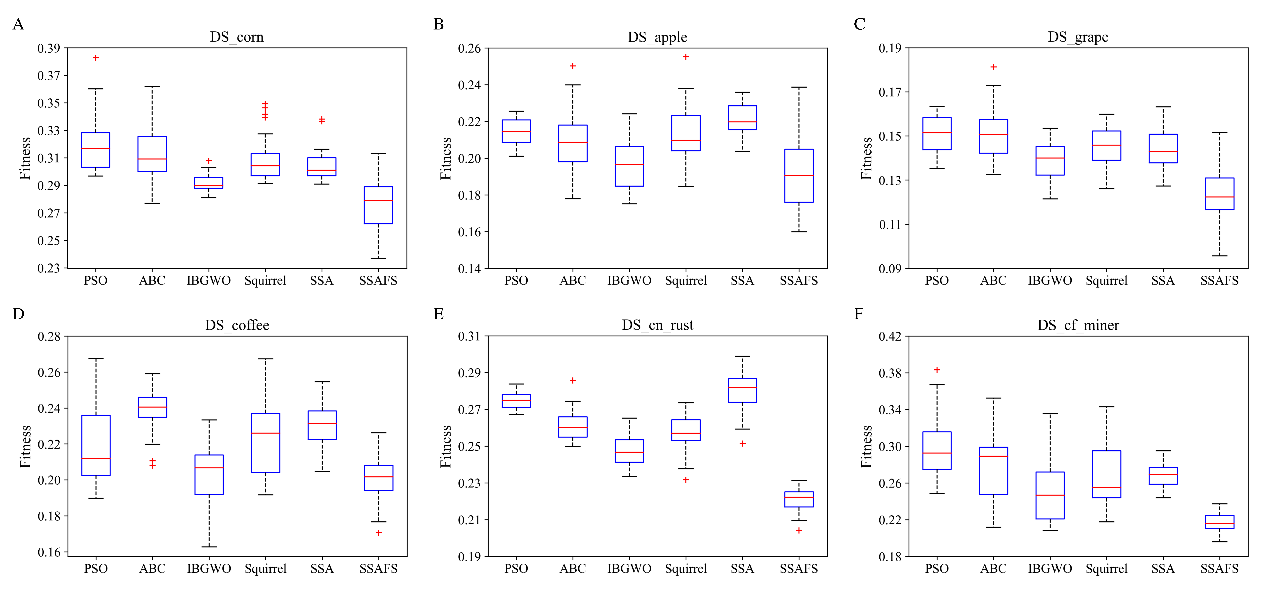


**Fig S6**. The distribution of the optimal solutions in the parallel computing on plant phenomics dataset (A) DS_corn; (B) DS_apple; (C) DS_grape; (D) DS_coffee; (E) DS_cn_rust; and (F) DS_cf_miner.
